# Prolonged Neuroinflammation after Lipopolysaccharide Exposure in Aged Rats

**DOI:** 10.1371/journal.pone.0106331

**Published:** 2014-08-29

**Authors:** Hui Qun Fu, Ting Yang, Wei Xiao, Long Fan, Yan Wu, Niccolò Terrando, Tian Long Wang

**Affiliations:** 1 Department of Anesthesiology, Xuanwu Hospital, Capital Medical University, Beijing, China; 2 Department of Physiology and Pharmacology, Karolinska Institutet, Stockholm, Sweden; 3 Department of Anatomy, Capital Medical University, Beijing, China; Imperial College London, Chelsea & Westminster Hospital, United Kingdom

## Abstract

Inflammation is a hallmark of several disease states ranging from neurodegeneration to sepsis but is also implicated in physiological processes like ageing. Non-resolving inflammation and prolonged neuroinflammation are unclear processes implicated in several conditions, including ageing. In this study we studied the long-term effects of endotoxemia, as systemic lipopolysaccharide (LPS) injection, focusing on the role of astrocyte activation and cytokine release in the brain of aged rats. A single dose of LPS (2 mg/kg) or 0.9% saline was injected intraperitoneally in aged rats. Levels of pro-inflammatory cytokines (TNFα and IL-1β) and NF-κB p65 activation were measured systemically and in hippocampal tissue. Astrocytes and cytokines release in the CNS were detected via double immunofluorescence staining at different time-points up to day 30. Serum levels of TNFα and IL-1β were significantly increased acutely after 30 minutes (p<0.001) and up to 6 hours (p<0.001) following LPS-injection. Centrally, LPS-treated rats showed up-regulated mRNA expression and protein levels of pro-inflammatory cytokines in the hippocampus. These changes associated with astrogliosis in the hippocampus dentate gyrus (DG), IL-1β immunoreactivity and elevated NF-κB p65 expression up to day 30 post LPS exposure. Overall, these data demonstrate that LPS induces prolonged neuroinflammation and astrocyte activation in the hippocampus of aged rats. Hippocampal NF-κB p65 and excessive astrocytes-derived IL-1β release may play a pivotal role in regulating long-lasting neuroinflammation.

## Introduction

Dysregulated inflammation is a hallmark of several disease states [1]. Systemic inflammation, for example as a result of infection or aseptic surgical trauma, can activate the innate immune system, launching a cascade of physiological and behavioral changes ultimately affecting the central nervous system (CNS) [2], [3]. Release of pro-inflammatory cytokines, namely tumor necrosis factor-alpha (TNF-α) and interleukin-1β (IL-1β), together with pathogen-associated molecular patterns (PAMPs) have been classically associated with symptoms of *sickness behavior*, which includes general fatigue, decreased food intake, fever, somnolence, and hyperalgesia [4]. Activation of the innate immune system and the ensuing pro-inflammatory milieu can also impair learning and memory processes in the CNS, contributing to the transient, longer lasting, and in some cases permanent cognitive dysfunction [5]. A role for pro-inflammatory cytokines and associated cognitive decline has been proposed in a variety of models, ranging from sepsis to surgery-induced cognitive decline and many neurodegenerative conditions [6].

Neuroinflammation is a critical pathological hallmark in neurodegenerative disorders and other CNS impairments, especially with advanced age [7]. Recent evidence suggests systemic inflammation is a pivotal component in mediating cellular senescence, changes in neuronal plasticity, neurogenesis, neuronal loss, and overall contributing to cognitive decline [8]. Advanced age is also a known critical risk factor for a number of clinical conditions, including dementia like Alzheimer’s disease (AD), postoperative cognitive decline, cancer and cardiovascular diseases [9], [10]. Furthermore, clinical studies suggest greater risks for geriatric patients in developing postoperative complications, including infections and delirium that often result in further comorbidities and higher mortality rates [11].

Lipopolysaccharide (LPS), a component of Gram-negative bacteria outer membrane wall, has been used as a classical model for activating immunocompetent cells and triggering a systemic and central inflammatory response. In models of LPS-induced neuroinflammation, microglia activation has been associated with persistent neuronal damage, changes in long-term potentiation and cognitive dysfunction [12]. Microglia, also regarded the resident macrophages in the CNS, actively survey and maintain homeostasis in the brain [13]. Although activation of these cells is a key component of several neurodegenerative conditions and contribute to behavioral and cognitive deficits [14], other glia-type cells including astrocytes also display pivotal roles in regulating CNS processes, synaptic plasticity and neuronal networking [15]. Yet, the contribution of these cell-types to the neuroinflammatory process is largely unexplored. In the present study we characterized the long-term neuroinflammatory response after a single systemic LPS injection and describe a critical role for long-lasting astrocyte activation in mediating hippocampal inflammation in aged rats. These results implicate a novel role for astrocytes, nuclear factor (NF)-kB activation and IL-1β signaling as possible targets for regulating neuroinflammation and senescence-associated secretory phenotype (SASP) during ageing.

## Materials and Methods

All experiments were conducted in accordance with the guidelines for experimental animal use and the protocol approved from the animal ethics committee of Capital Medical University. All efforts were made to minimize the number and suffering of the experimental animals.

### Animals and treatments

Male rats (Wistar, 20 months old, 500–700 g) were selected for the study and housed 2–3 per cage in controlled laboratory conditions (23°C on a 12 hr light/dark cycle with food and water *ad libitum*).

Rats were injected intraperitoneally (i.p.) with 2 mg/kg *Escherichia Coli* bacterial lipopolysaccharide (LPS) (serotype 055:B5, Sigma, St Louis, MO, USA) dissolved in 0.9% sterile saline, same volume of saline was used in control group. Rats were terminated at 0.5, 2, 6 hours and 1, 3, 7, 30 days after LPS injection (n = 8/group).

### Tissue preparation

On the termination day, animals were anesthetized by isoflurane (Forene, Abbott Laboratories, Queensborough, UK) and blood was taken from inferior vena cava. After 2 h of clotting at 4°C, the blood sample was centrifuged for 20 mins at 1000 g, the serum was collected and stored at −80°C.

After the blood draw, the animals (n = 4/group) were decapitated and brains were immediately removed and dissected on ice-cold frosted plate. The hippocampi were homogenized in ice cold lysis buffer (50 mM Tris –Hcl, 0.25 M sucrose, 2 mM EDTA, 10 mM EGTA, 1% Triton ×100, all from Sigma, St Louis, MO, USA) or RIPA Extraction Buffer (1% NP-40, 0.5% sodium deoxycholate, 0.1% SDS, all from Sigma, St Louis, MO, USA) with 1:100 protease inhibitor cocktail (Roche Diagnostics, Indianapolis, IN) and centrifuged at 10×5 g for 40 min at 4°C. The supernatants were collected and stored at −80°C for later analysis.

From another cohort (n = 4/group) of rats, brains were embedded in optimum cutting temperature (OCT) compound (Sakura Finetek USA, inc., Torrance, CA, USA) and snap frozen in liquid nitrogen. Tissues were then stored at −80°C for immunohistochemistry.

### ELISA measurement

IL-1β, TNF-α and NF-κB p65 in serum or hippocampus were measured with a rat IL-1β, TNF-α (R&D Systems, Inc. Minneapolis, MN, USA) or NF-κB p65 ELISA kit (Enzo Life Science, Farmingdale, NY, USA) following the manufacturers’ protocol. The protein extracted from hippocampus was quantified with BCA protein assay reagent kit (Pierce Biotechnology, Inc., Milwaukee, WI, USA).

### Real-time quantitative reverse transcriptase polymerase chain reaction (RT-PCR)

Total RNA was extracted from hippocampus using Trizol (Invitrogen, Paisley, UK) and purified with RNeasy Mini Kit (Catalog number 74104, Qiagen, Palo Alto, CA). The integrity of total RNA was measured by agarose gel electrophoresis and cDNA was synthesized with High Capacity cDNA Reverse transcription Kit according to the manufacturer’s protocol (Catalog number 4368814, Applied Biosystems). Primer sequences and amplification profiles used for TNF-α, IL-1β, NF-κB p65 and β-actin are described in Table 1. DNA amplification was carried out in 1×Taq polymerase buffer, 1.5 mM MgCl_2_ supplemented with 0.2 mM dNTPs, 25 pmol of 5′ and 3′ specific primers and 2 units of Taq polymerase in a volume of 50 µl. The mixture was amplified for 40 cycles (each cycle consisting of denaturation for 1 min at 94°C, annealing for 1 min 58°C, extension for 1 min at 72°C). Amplification products were separated on 1.8% agarose gel, and the intensity of each band was quantified using NIH Image software and expressed in arbitrary units (version 1.60).

**Table 1:** Sequences of Primers for RT-PCR.

	Forward Primers (5′→3′)	Reverse Primers (5′→3′)
TNF-α	CAGAGCAATGACTCCAAAGTA	CAAGAGCCCTTGCCC TAA
IL-1β	CACACTAGCAGGTCGTCATCATC	ATGAGAGCATCCAGCTTCAAATC
NF-κB p65	CTGCGATACCTTAATGACAGCG	CTGCGATACCTTAATGACAGCG
β-actin	TTTGAGGGTGCAGCGAACTT	ACAGCAACAGGGTGGTGGAC

### Immunofluorescence and microscopy

Coronal hippocampal sections were cut at 15 µm thick with freezing microtome (CM1850, Leica Microsciences, Mannheim, Germany). The sections of the hippocampus were selected according to anatomical landmarks corresponding to the atlas of Paxinos and Watson between Bregma −2.30 and Bregma −3.60 for immunofluorescence staining. Sections were then fixed in ice-cold 4% paraformaldehyde (catalog number CM-0055, Lifeline Cell Technology, Walkersville, MD) for 15 min, permeabilized with 0.3% Triton X-100 (Sigma, St Louis, MO, USA) and blocked in 3% horse serum (Invitrogen, New Zealand). Sections were then incubated with primary antibodies overnight at 4°C: goat anti-TNF-α IgG (1:100; catalog number AF-510-NA, R&D, Systems, Inc., Minneapolis, MN, USA), goat anti-IL-1β IgG (1:100; catalog number AF-501-NA, R&D, Systems, Inc., Minneapolis, MN, USA), rabbit anti-NF-kB p65 IgG (1:100; catalog number Ab7970, Abcam, Cambridge, MA, UAS), and mouse anti-Glial fibrillary acidic protein (GFAP) IgG (1:1000; catalog number MAB360 Millipore, Billerica, MA, USA) respectively. After washing, sections were incubated with Alexa Fluor 594 donkey anti-Rabbit IgG (1:500; catalog number: A21207, Invitrogen, Paisley, UK), Alexa Fluor 488 donkey anti-Mouse IgG (1:500; catalog number: A21202, Invitrogen, Paisley, UK), Alexa Fluor 594 donkey anti-goat IgG (1:500; catalog number: A11058, Invitrogen, Paisley, UK) for 2 h in dark at room temperature. Negative control sections were applied with the same procedures without the presence of primary and secondary antibodies. All the sections were counterstained with Hoechst 33342 (1:1000; Roche, Mannheim, Germany). Samples were analyzed with confocal microscopy (Leica TCS2, Leica, Benshein, Germany) and image analyzing system (Optimas 6.5, CyberMetrics, Scottsdale, AZ) by an investigator blinded to the experimental groups. Cell counts were performed within 1 mm diameter in DG region, 10 sections were evaluated for each animal. The percentages of double positive stained cells (GFAP/TNF-α, IL-1β or NF-κB) in total GFAP+ cells were calculated. Mean gray levels on the ipsilateral and contralateral side were measured in 10 sections under identical conditions in DG region. The average gray values for GFAP staining were obtained with the NIH ImageJ software.

### Statistical analysis

Data analyses were performed with SPSS software (Ver. 11.5, IBM, Chicago, IL, USA). Statistical significance was determined by one way of ANOVA followed by Bonferroni *post hoc* analysis wherever appropriate. A value of *p*<0.05 was considered statistically significant. Pearson’s correlation coefficient was used for correlation analyses.

## Results

### LPS rapidly increases systemic TNF-α and IL-1β

**Figure 1:**
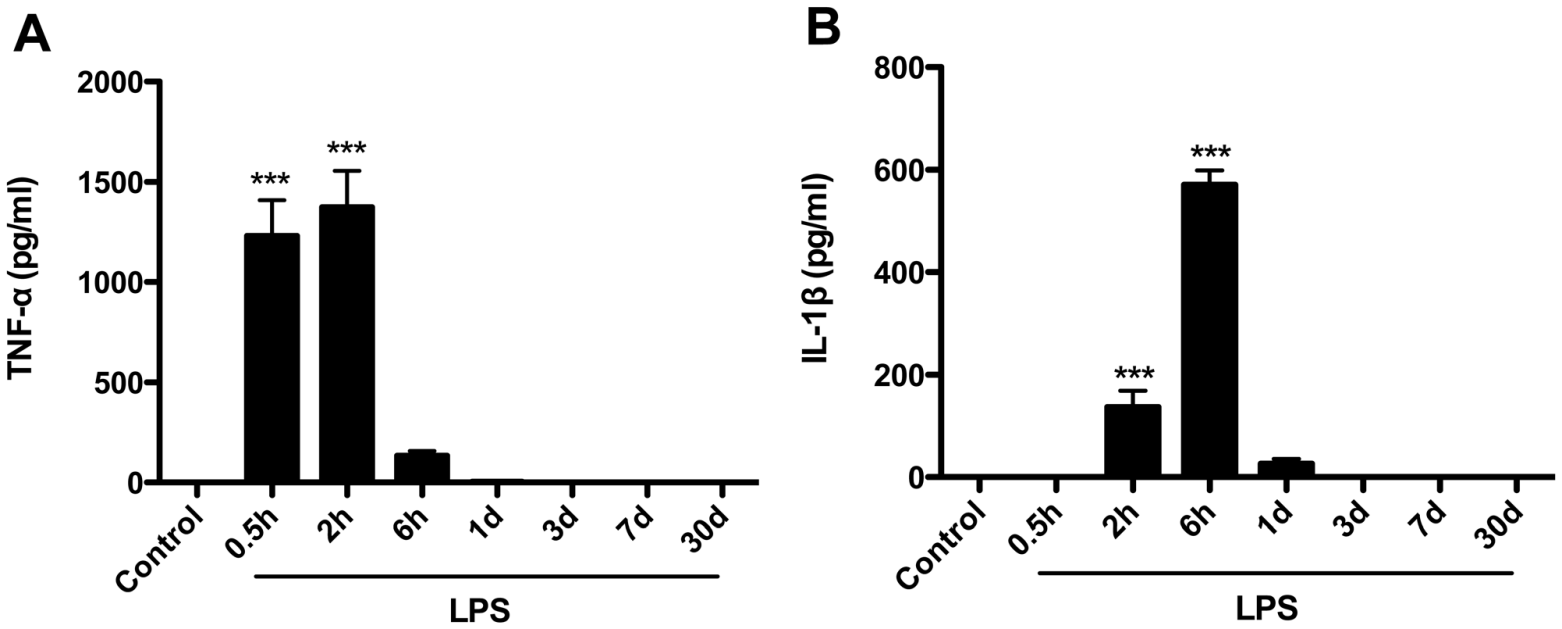
LPS increase systemic levels of TNF-α and IL-1β in aged rats. Animals were adminstrated either LPS (2 mg/kg) or equal volume of saline (i.p.). No significant changes were measured in saline injected animals at all time points. (A) TNF-α in serum significantly increased at 0.5 h and 2 h after LPS administration, and returned to control level at day 1. (B) There was also a siginificant increase in circulating IL-1β level following LPS injection but this was delayed from TNF-α release and started at 2 h. Data are expressed as mean ± standard error of the mean (n = 8) and compared by 1-way analysis of variance followed with Boferroni *post hoc* analysis, ***p*<*0.001 *vs* Control.

To assess the systemic effect of LPS we measured classical pro-inflammatory cytokines in serum. Endotoxin induced a strong, but transient, systemic inflammatory responses in the aged rats. After LPS exposure, circulating TNF-α levels rapidly and significantly increased already at 0.5 hours (1231±178.1 pg/ml, p<0.001), peaking at 2 hours (1374±181.7 pg/ml, p<0.001) and returning to baseline by day 1 (Figure 1A). The increase of IL-1β in serum was delayed from the TNF-α response, increasing after 2 hours (136.7±32.1 pg/ml, p<0.001) with circulating values remaining detectable up to day 1 (26.63±9 pg/ml), returning to baseline thereafter (Figure 1B).

### LPS alters pro-inflammatory cytokines and NF-κB activity in the hippocampus

**Figure 2:**
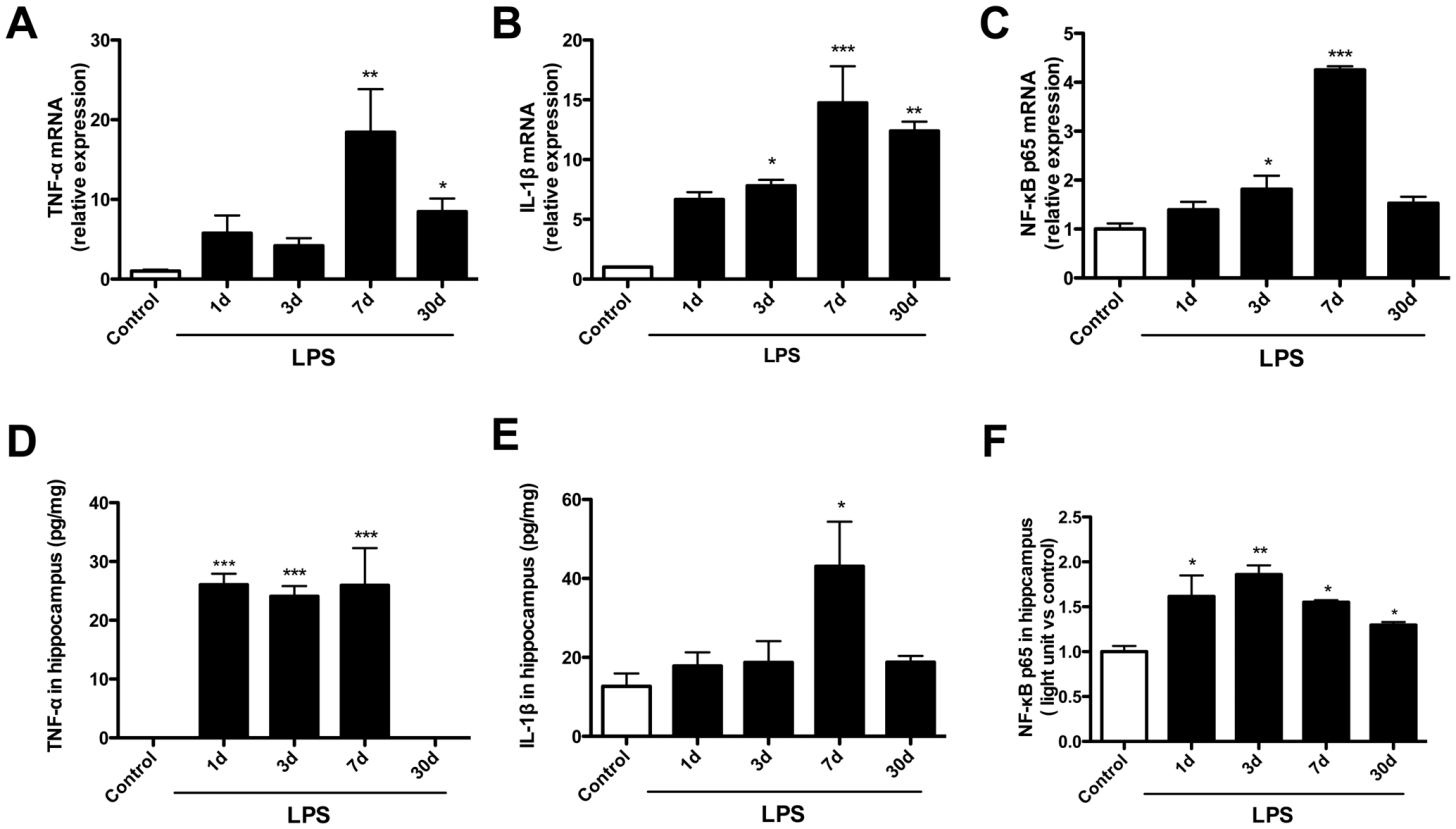
Gene expression and protein level of TNF-α, IL-1β and NF-κB in the hippocampus. After LPS injection the expression of pro-inflammatory cytokines including TNF-α (A), IL-1β (B) and NF-κB p65 (C) mRNA activity were measured at different time points up to day 30. (A) Levels of TNF-α peaked at day 7 (18.4±5.4) and remained mildly, but significnalty, increased at day 30 (8.5±1.6). (B) IL-1β started to be up-regualted at day 3 (7.8±0.5), peaked at day 7 (14.7±3.1) and remained increased at mRNA level up to day 30 (12.4±0.8). (C) NF-κB p65 expression was found increased at day 3 (1.8±0.3) and day 7 (4.3±0.1) post LPS exposure. Protein levels were also measure using ELISA (D–E–F). (D) TNF-α levels in hippocampal homogenate were stably increased from day 1 to day 7. (E) IL-1β was detected only at day 7 with other values comparable to saline injected rats. (F) NF-κB p65 was up-regulated at all time-points, from day 1 to day 30. Data are expressed as mean ± standard error of the mean (n = 4) and compared by 1-way analysis of variance followed with Boferroni *post hoc* analysis, *p<0.05, **p<0.01, ***p<0.001 *vs* Control respectively.

To investigate the remote effects of LPS on the ageing brain we measured gene expression and protein levels of molecules critically involved in innate immune signaling. Both mRNA expression and protein levels of TNF-α, IL-1β, and NF-κB were significantly elevated in the hippocampus after systemic LPS compared to controls. Notably, mRNA expression of TNF-α, IL-1β, and NF-κB peaked 7 days after LPS exposure (p<0.01, p<0.001, p<0.001 respectively, Figure 2A–C) and, for TNF-α and IL-1β, levels remained up-regulated up to day 30 (p<0.05, p<0.01, respectively).

Protein levels were also detected by ELISA in hippocampal homogenate. TNF-α was found elevated from day 1 (26.0±1.9 pg/mg, p<0.001) to day 7 (25.9±6.3 pg/mg, p<0.001, Figure 2D) whereas IL-1β only peaked at day 7 (43.1±11.3 pg/mg, p<0.05) with levels remaining at baseline otherwise (Figure 2E). NF-κB activity was also up-regulated at all time points up to day 30 after LPS injection (1d: 1.6±0.2, 3d: 1.9±0.1, 7d: 1.6±0.02, 30d: 1.3±0.03, p<0.05 and p<0.01 respectively), as compared with after saline injection (Figure 2F).

### Hippocampal astrogliosis and sustained cytokines after LPS

**Figure 3:**
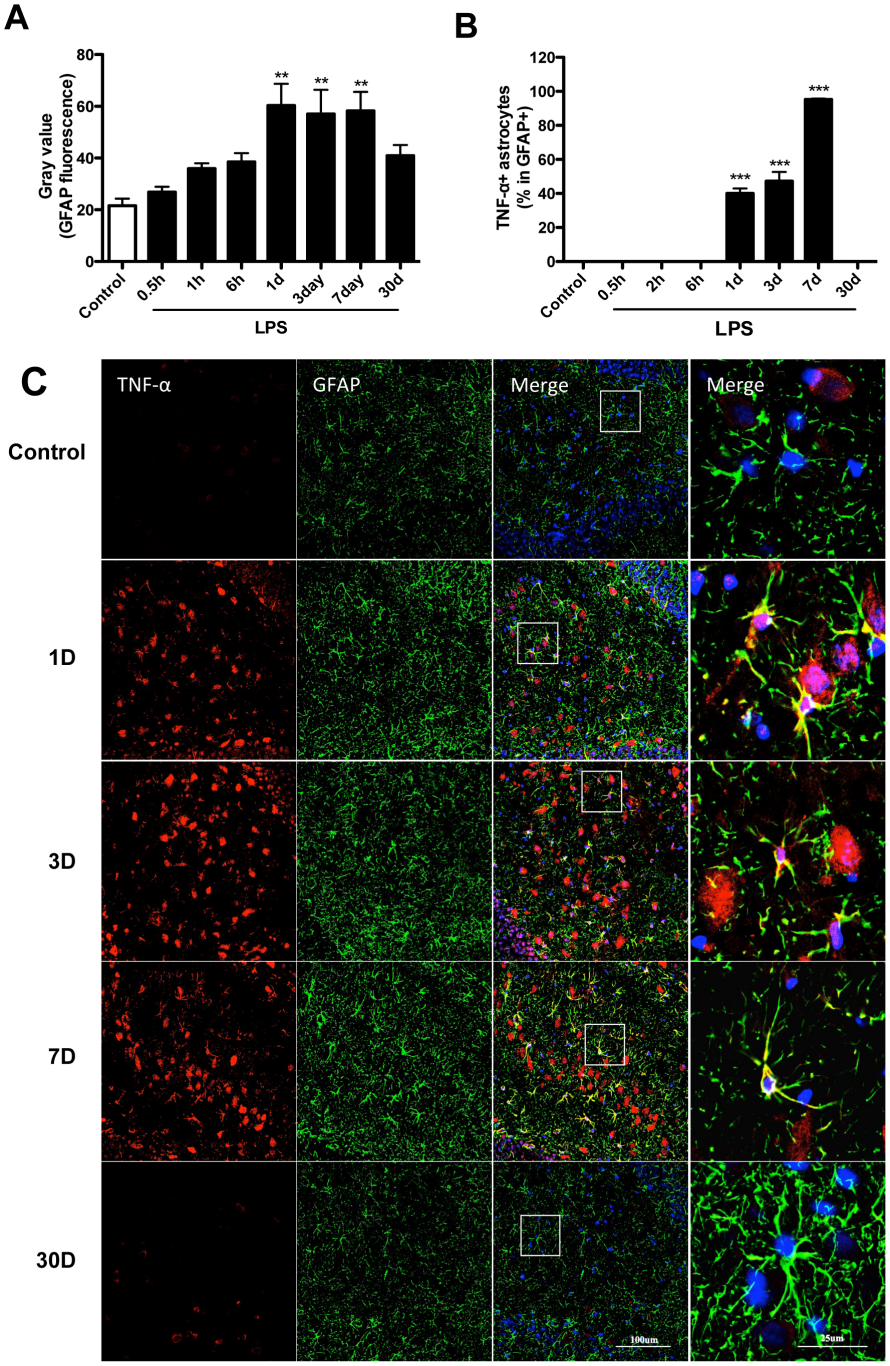
Astrocytes activity and GFAP/TNF-α double staining in the DG area. Astrocyte activity was measure with GFAP immunofluorescence. Representative photomicrographs show control and LPS-injected rats at different time points up to day 30. (A) Densitometry of GFAP fluorescence intensity was up-regulated from day 1 to day 7. Following LPS injection, astrocytes showed changes in morphology, which was recognized by decreased ramification and hypertrophy of the soma. (B) Double fluorescence staining of GFAP and TNF-α in hippocampal astrocytes. The ratio of TNF-α positive astrocytes in total astrocytes (GFAP-positive cells) was significantly increased from day 1 and peaking on day 7. Pictures show DG area, data are expressed as mean ± standard error of the mean (n = 4) and compared by 1-way analysis of variance followed with Boferroni *post hoc* analysis, **p<0.01, ***p<0.001 *vs* Control.

Although LPS-induced neuroinflammation has been pivotally associated with microglia activation, astrocytes play an important role in maintaining brain homeostasis and protecting surrounding neurons from damage like infective agents [15]. One day after LPS injection, astrocytes in dentate gyrus (DG) showed activated morphology characterized by decreased ramifications and hypertrophy of the soma, as measured by GFAP immunofluorescence. Densitometric analysis of the fluorescence intensity showed higher intensity of GFAP intermediated filaments staining from day 1 up to day 30 after LPS injection (p<0.05 respectively, Figure 3A).

We used double immunofluorescence labeling to investigate the cellular origin of the pro-inflammatory cytokines increased in the hippocampus after LPS. A positive correlation in TNF-α and GFAP-positive was noted in astrocytes, with a time dependent activation from day 1 to day 7 (p<0.001 respectively, Figure 3B). Furthermore, this was consistent with the TNF-α protein increase found in the hippocampus at the same time points (Figure 2D).

**Figure 4:**
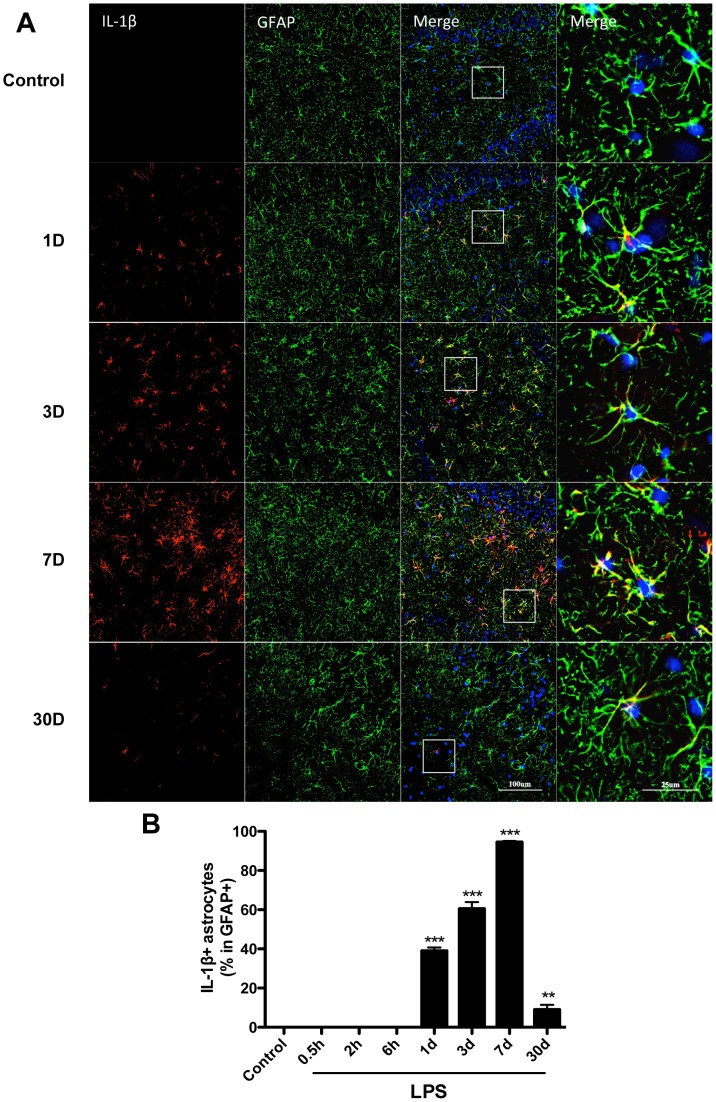
Sustained IL-1β up-regulation in hippocampal astrocytes. IL-1β immunoreactivity was measured in GFAP-positive cells after LPS exposure or saline. Levels of IL-1β were found up-regulated from day 1 up to day 30. Higher magnification insets highlight the co-localization of IL-1β with GFAP (A). The ratio of IL-1β positive astrocytes in total astrocytes (GFAP positive cells) was quantified (B). Pictures show DG area, data are expressed as mean ± standard error of the mean (n = 4) and compared by 1-way analysis of variance followed with Boferroni *post hoc* analysis, **p<0.01, ***p<0.001 *vs* Control.

Next, we measured IL-1β expression and found a gradual increase in GFAP/IL-1β-positive cells from day 1, with levels peaking on day 7 and retaining some residual activity up to day 30 (p<0.001, p<0.01 respectively, Figure 4). This further corroborates the significant up-regulation of IL-1β in hippocampus homogenate we measured by ELISA on day 7 (Figure 2E), however using immunofluorescence we also detected some IL-1β-positive cells up to day 30 after LPS exposure (p<0.01, Figure 4B), which is consistent with elevated mRNA expression (Figure 2B). Taken together, these results also highlight a key role of other cell-types or subpopulations, including neurons and microglia, in contributing to the overall neuroinflammatory response after LPS exposure.

### NF-κB p65 signaling is disrupted in hippocampal astrocytes

**Figure 5:**
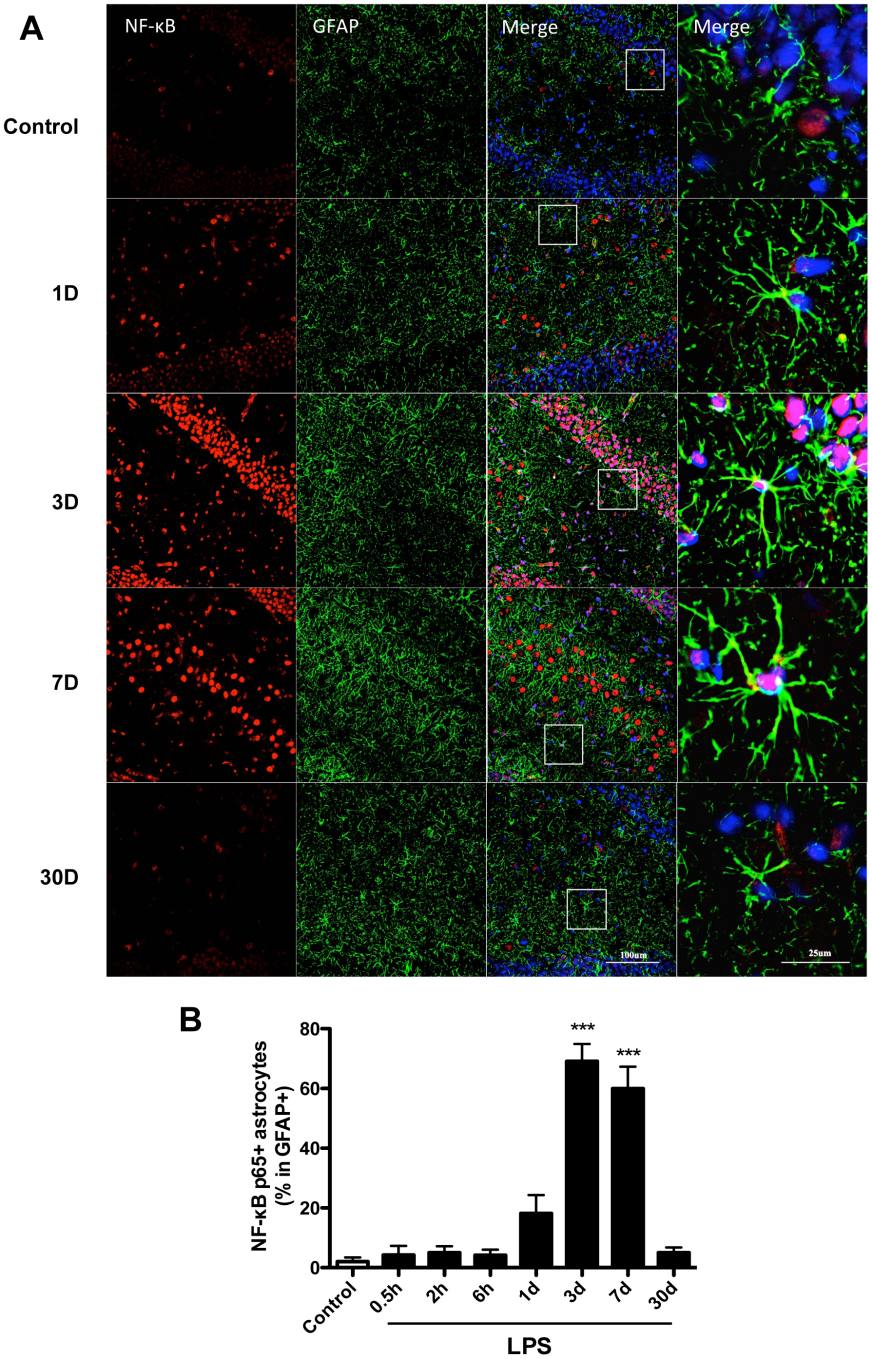
NF-κB p65 DNA biding activity in astrocytes after LPS. NF-κB activation in astrocytes was measured by NF-κB p65/GFAP double staining. Confocal images and quantification show a gradual increase in NF-κB p65 activity and nuclear translocation from day 1 to day 7, with a peak on day 3. Representative high magnification pictures are shown in the insets. Pictures are representative from DG area, data are expressed as mean ± standard error of the mean (n = 4) and compared by 1-way analysis of variance followed with Boferroni *post hoc* analysis, ***p<0.001 *vs* Control.

To test if LPS engaged NF-κB signaling as a mechanism to release cytokines and contribute to neuroinflammation we evaluated the level of activated NF-κB p65 in hippocampal astrocytes (Figure 5). In the control group the proportion of NF-κB p65 positive astrocytes in total astrocytes in the hippocampus was lower (5±2.3%) at all time points. However, after LPS exposure this proportion reached 18.2±6.1% at day 1, peaking at day 3 (69.1±5.8%, p<0.001) and maintained high levels up to day 7 (60.0±7.3%, p<0.001). At these time-points, LPS-activated astrocytes also showed evident nuclear translocation. No cytokines expression was detected in controls and during the acute phase response (0.5–6 hours) after LPS injection (Figure 3–5).

## Discussion

This study demonstrates that a single injection of LPS, mimicking a systemic infection as often occurs in older adults, causes long-lasting neuroinflammatory changes. Reactive astrocytes may play a critical role in sustaining this neuroinflammatory response following systemic LPS injection in aged rats together with other neuro-immune cell types. The mechanisms for this prolonged CNS dysfunction involve sustained release of pro-inflammatory cytokines in the hippocampus and, partially, NF-κB activation in astrocytes.

Long-lasting inflammation, also referred to as non-resolving inflammation, significantly impairs organ integrity and is a major driver of tissue damage [1]. Cytokines mediate many of the physiological and behavioral changes after infection or trauma, both in humans and animals, and become dysregulated in multiple disease states [16]. The mechanisms whereby cytokines and other pro-inflammatory mediators affect the brain and CNS function remain largely unexplored, especially in correlation with advanced age. In this study after a single systemic LPS injection the expression of TNF-α in the serum preceded the up-regulation of IL-1β (Figure 1B) suggesting that activation of the innate immune system and release of pro-inflammatory cytokines in the systemic circulation negatively affects CNS function. Cytokines and alarmins like high-mobility group box 1 (HMGB-1) directly impair endothelial function disrupting tight junctions and contributing to the blood-brain barrier (BBB) opening during infection and trauma [17]–[19]. TNF-α has been previously reported as an early marker for mediating cognitive decline after peripheral sterile injury [3] contributing to the BBB opening during systemic inflammation and neurodegeneration [20], [21]. Notably, LPS also has the ability to disrupt immune–endothelial interaction at the BBB, thus allowing pro-inflammatory immune cells to directly infiltrate into the CNS [22].

After LPS administration we found a delayed increase in levels of TNF-α and IL-1β in the hippocampus, starting at day 1 post-exposure and persisting up to day 30 (Figure 2). IL-1β is the prototypic pyrogen and has been described to play a key role both in endotoxemia and trauma-mediated cognitive dysfunction [23], [24]. Moreover, modulation of the IL-1 response with the administration of the naturally occurring interleukin-1 receptor antagonist (IL-1Ra) significantly improved neuroinflammation and neurological outcome in animals with multiple risk factors for stroke, including advanced age [25]. Neuroinflammation has become a critical pathological hallmark in several conditions, yet its role in disease onset and progression remains debated and controversial. In our study we focused on astrocytes activation after LPS exposure and looked into long-term cellular interactions in the hippocampus.

Astrocytes are the most abundant glia-type cells of the CNS, comprising at least one third of human brain cells, and their functions in regulating neuronal processes and the brain microenvironment are not fully understood [26]. Astrocytes display several immune functions and the increasing expression of GFAP has been noted during ageing, with significant changes after the age of 65 years in the hippocampus of human postmortem tissue [27]. Ageing is characterized by the establishment of a chronic pro-inflammatory environment also termed “inflamm-aging” [28]. Soluble signaling mediators, including cytokines like IL-1β, IL-6 and chemokines are secreted at high levels during ageing and in senescent cells [29]. Chronic inflammation and this pro-inflammatory milieu have been correlated with loss of cell functionality, dysfunctional neurogenesis, impaired synaptic plasticity and neuronal loss as also seen in many neurodegenerative conditions [30]. The ageing brain displays several hallmarks of the senescence-associated secretory phenotype (SASP) and astrocytes are important regulators of the process of cellular senescence during normal ageing (reviewed in [31]). After LPS treatment, astrocytes activation was noted in the DG area of the hippocampus (Figure 3A, C) and GFAP/IL-1β immunoreactivity remained significantly up-regulated up to day 30 (Figure 4). This was further corroborated at mRNA level (Figure 2B) and suggests a possible role for IL-1 signaling in sustaining chronic inflammation in the CNS [32]. It is possible that this prolonged astrocyte reactivity associates with non-resolving inflammation and SASP in the aged and vulnerable brain. Following endotoxemia microglia also become activated and release pro-inflammatory cytokines, including IL-1β [33]. However, LPS-triggered reactive astrocytes have been associated with detrimental effects to synaptic plasticity and neuronal function, for example through the activation of the complement cascade and reduced production of neurotrophic factors [34]. In our study we found activation of NF-κB p65 in hippocampal astrocytes (Figure 5), suggesting the presence of pro-inflammatory signaling in these cells after LPS exposure. NF-κB is a pivotal regulator of the inflammatory-resolving switch and its activation and nuclear translocation is a crucial component in modulating pro-inflammatory cytokines and neuroinflammation [35]. In this model we noted an important role for pro-inflammatory cytokines in mediating short and long-term neuroinflammatory changes dependent both on GFAP activity in the hippocampus and NF-κB signaling. Although microglia have been classically regarded as the immunocompetent cells in the CNS and associated with neuroinflammation in several neurological conditions [36], astrocytes also express innate immune pattern-recognition receptors (PRRs) on their surface and display important functions in regulating neuroinflammation and SASP through NF-κB and C/EBP transcription factor signaling [37], [38].

The hippocampus is critical for memory processing and LPS has been previously shown to induce both acute and chronic behavioral changes, including memory impairments, neurodegenration and cognitive decline [39], [40]. Neurocognitive assessment was not performed in our study but the prolonged astrogliosis and cytokine release in the hippocampus suggest possible interference with learning and memory processes. Recently, an association between reactive astrocytes and synaptic plasticity was reported following systemic inflammation after orthopedic trauma [41]. Furthermore, in the aged brain peripheral infection caused exaggerated and prolonged expression of pro-inflammation cytokines in the hippocampus (DG and CA1), corresponding to a disruption in working memory [42].

## Conclusion

Overall, these results indicate that astrocytes, amongst neurons and other immunocompetent cells in the CNS, are critically involved in the development of neuroinflammation following acute peripheral infection in aged rats. Failure to switch off NF-κB activity in the aged brain, thus perpetuating the pro-inflammatory environment, may represent the substrate for non-resolved inflammation in the CNS. Whether targeting NF-κB activity in astrocytes and neurons improves neurological outcome remains an important question for future study.
